# Anatomical lesion extent and recurrent failure after balloon angioplasty in arteriovenous fistulas: a competing-risk analysis

**DOI:** 10.3389/fcvm.2026.1906106

**Published:** 2026-08-12

**Authors:** Kelong Shi, Jiang Xu, Yongxiang Tang, MingXu Ou, GuChao Zhou

**Affiliations:** 1 Bengbu Medical University, Bengbu, Anhui, China; 2 Tongling People's Hospital, Tongling, Anhui, China

**Keywords:** arteriovenous fistula, competing risk, digital subtraction angiography, percutaneous transluminal angioplasty, re-failure

## Abstract

**Background:**

Risk stratification for arteriovenous fistula (AVF) re-failure after percutaneous transluminal angioplasty (PTA) relies on systemic comorbidity markers. These markers are costly and inconsistently predictive in resource-limited settings. We hypothesized that intraoperative digital subtraction angiography (DSA) variables, lesion length and lesion morphology, could stratify the risk of re-failure without additional testing.

**Methods:**

This single-center retrospective derivation study included 243 patients with successful PTA using uniform Mustang™ balloons. We used Fine-Gray subdistribution hazard models with death and kidney transplantation as competing events. Missing data were handled by multiple imputation. Model discrimination and calibration were assessed by optimism-corrected C-index via 500 bootstrap resamples and calibration plots. Lesion length was modelled with a restricted cubic spline (3 knots); lesion location was analysed as four mutually exclusive categories with anastomotic/peri-anastomotic stenosis as the reference. Calibration was assessed in quintiles of predicted 1-year cumulative incidence.

**Results:**

During follow-up, 103 patients (42.4%) experienced re-failure and 24 (9.9%) had competing events. Lesion length showed a strong non-linear association with re-failure (test for non-linearity, *P* < 0.001). Relative to the median length of 1.75 cm, the subdistribution hazard ratio (SHR) was 0.25 (95% CI, 0.20–0.32) at 1 cm, 1.46 (95% CI, 1.37–1.56) at 2 cm and 4.31 (95% CI, 3.37–5.51) at 3 cm. Once lesion length was modelled as a spline, the effect of composite morphology was attenuated by 35%, from 2.81 (95% CI, 1.70–4.66) under a linear specification to 1.83 (95% CI, 1.13–2.97) (*P* = 0.014). The final model achieved an optimism-corrected C-index of 0.79 at 1 year and 0.80 at 2 years. Age, diabetes and coronary artery calcium score showed no significant association.

**Conclusion:**

Intraoperative DSA-derived lesion length showed a strong, markedly non-linear association with AVF re-failure after PTA and carried nearly all of the discriminative ability of the model; the apparent effect of composite morphology was substantially attenuated once length was modelled appropriately, indicating that morphology acts in part as a surrogate for lesion extent. Because vessel diameter, stenosis severity, local calcification and procedural parameters were not available, these findings are hypothesis-generating and require prospective external validation with comprehensive lesion characterisation before any clinical implementation.

## Introduction

Durable vascular access is the cornerstone of sustainable hemodialysis. The autogenous arteriovenous fistula (AVF) is universally preferred for its superior patency, lower infection risk, and reduced mortality compared with prosthetic grafts or central venous catheters (1). When flow-limiting stenosis develops, percutaneous transluminal angioplasty (PTA) is the first-line intervention per the 2019 KDOQI update (1). Yet technical success does not guarantee durable patency: 40%–60% of patients require repeat intervention within the first year (2), and in patients with inflow arterial stenosis, cumulative re-intervention rates exceed 70% by the second year (3). Against this backdrop of high recurrence, identifying high-risk patients at the bedside, thereby enabling intensified postoperative surveillance and earlier secondary intervention, carries substantial clinical and health-economic value.

Anatomical factors, particularly lesion length, have emerged as independent predictors of restenosis, with lesions exceeding 2 cm associated with significantly poorer patency (4). The underlying pathophysiology involves neointimal hyperplasia driven by smooth muscle cell proliferation, extracellular matrix deposition, and endothelial dysfunction (5). These processes are primarily governed by local anatomical severity, yet may be independently modulated by systemic vascular burden such as aortic arch calcification. Contemporary risk-stratification paradigms, however, rely predominantly on patient-level systemic markers such as age, diabetes mellitus, and coronary artery calcium (CAC) scores (6, 7). Yet accumulating evidence from observational studies and meta-analyses suggests these conventional markers exhibit only modest and inconsistent predictive discrimination for post-PTA patency, with hazard ratios often hovering near unity (6, 7).

Intraoperative digital subtraction angiography (DSA) is performed in virtually every PTA procedure to guide balloon placement and confirm angiographic success. It captures granular local anatomy, lesion length, exact location, and morphological pattern, that directly reflects structural disease severity. Despite this rich information, DSA images are rarely exploited for longitudinal risk prediction. Worldwide, more than half of dialysis patients in low-income regions lack access to routine laboratory monitoring, underscoring the need for zero-cost, anatomy-based risk stratification tools (8). Hemodynamic studies further demonstrate that geometric factors and flow patterns at the anastomosis influence stenosis development, supporting the biological plausibility of DSA-derived predictors (9).

We therefore posed a pragmatic, clinically grounded question: in grassroots dialysis centers where advanced laboratory platforms and specialized imaging are scarce, can two routine intraoperative DSA variables, lesion length and composite morphology, provide preliminary, zero-additional-cost risk stratification for re-failure after PTA, without reliance on systemic workup?

## Materials and methods

### Study design and population

This retrospective derivation study extends a parent cohort on AVF maturation and failure (currently under major revision). Of the 645 patients enrolled in the parent cohort, the present study includes 243 patients (37.7%) who developed first-time flow-limiting stenosis or thrombosis after successful AVF maturation and underwent successful PTA (Supplementary Figure S1). The parent cohort focuses on predictors of primary AVF maturation and first-time failure, whereas the present study specifically examines intraoperative DSA variables as predictors of re-failure after successful PTA, which represents a distinct hypothesis, outcome, and analytical framework. The parent study consecutively enrolled 645 end-stage renal disease patients undergoing first-time radial-cephalic AVF creation between January 2022 and January 2024. Among 568 patients who achieved maturity, defined as successful cannulation with blood flow ≥250 mL/min sustained for at least four consecutive sessions, 277 developed first-time flow-limiting stenosis or thrombosis and underwent PTA. We applied three exclusion criteria: intraoperative PTA failure (residual stenosis >30% after maximal balloon inflation); unsalvageable thrombosis within 30 days post-PTA without fistula reuse; and permanent access loss before two successful post-PTA dialysis sessions. After exclusions, 243 patients remained. The index date was the day of first successful PTA.

All procedures were performed in a single hybrid angiography suite by three attending vascular surgeons. As a grassroots center without reimbursement for advanced endovascular devices, all PTA procedures used Mustang™ balloon catheters (Boston Scientific, Marlborough, MA, USA), plain, non-coated balloons without cutting or scoring elements. This uniform device use eliminates device-type confounding, ensuring that observed outcome heterogeneity reflects patient anatomy and disease biology rather than inter-procedural technological variation.

### Outcome definitions

The primary outcome was re-failure after successful PTA, defined as a composite of: recurrent stenosis requiring repeat PTA or surgical revision; thrombosis confirmed by DSA or duplex ultrasound; or clinical failure (fistula no longer usable for dialysis). For patients without repeat intervention but suspected of failure, we required objective documentation of two consecutive dialysis sessions with blood flow <200 mL/min or incomplete prescribed dialysis, plus confirmatory imaging showing >50% restenosis. This dual requirement minimized misclassification due to puncture-related flow problems or temporary hematoma.

Competing events were all-cause death (*n* = 21) and kidney transplantation (*n* = 3). We deliberately did not treat permanent fistula abandonment as a competing event, as abandonment is almost always a downstream consequence of confirmed re-failure rather than an independent precluding event. Right-censoring included survival with functional fistula at the study cutoff (December 31, 2025), loss to follow-up (*n* = 9, 3.7%), and transfer with unobtainable information.

### Predictors

All anatomical predictors were extracted from intraoperative DSA images acquired immediately before balloon inflation. Lesion length was measured along the centerline of the stenotic segment from proximal to distal shoulder, recorded in centimeters. Lesion location was classified into four mutually exclusive categories: (i) anastomotic or peri-anastomotic (stenosis confined to the anastomosis and adjacent 1-cm segment); (ii) arterial inflow (proximal to the anastomosis); (iii) venous outflow (distal to the anastomosis); and (iv) composite, defined as multi-segment stenosis spanning the anastomosis and at least one adjacent segment, with stenotic segments separated by ≥1 cm of relatively normal vessel (diameter stenosis <30%). This category was specifically designed to capture skip lesions rather than simple long-segment continuous stenosis.

We prespecified that composite stenosis and lesion length might exhibit partial operational overlap. To ensure both variables could legitimately coexist in the same model, we planned variance inflation factor (VIF) assessment and an interaction term test. Systemic markers, age at index PTA, diabetes status, and CAC score (quantified by AI-assisted software from preoperative non-contrast chest CT using the Agatston method, categorized as 0, 1–100, or >100 Agatston units),served as reference predictors against which anatomical variables were benchmarked. All four categories were retained as distinct levels in every multivariable model. Anastomotic/peri-anastomotic stenosis (*n* = 92) served as the reference category, and arterial inflow (*n* = 29), venous outflow (*n* = 67) and composite lesions (*n* = 55) were each represented by a separate indicator variable.

### Statistical analysis

We used Fine-Gray subdistribution hazard models to estimate the effect of predictors on the cumulative incidence of re-failure, treating death and kidney transplantation as competing events (10). The primary model included lesion length, modelled with a restricted cubic spline with three knots placed at the 10th, 50th and 90th percentiles of its distribution (0.21, 1.75, 7.05 cm), and lesion location as four mutually exclusive categories with anastomotic/peri-anastomotic stenosis as the reference. Non-linearity was formally tested by a Wald test on the spline terms, and subdistribution hazard ratios are reported relative to the median lesion length of 1.75 cm. To evaluate independence from systemic disease burden, we fitted a multivariable model adding age, diabetes, and CAC category.

The proportional subdistribution hazards assumption was assessed via covariate-by-log-time interactions; significant slopes (*P* < 0.05) were interpreted as evidence of non-proportionality, prompting time-varying coefficient models. Missing CAC data (*n* = 8, 3.3%) were handled by multiple imputation with chained equations (m = 20 datasets), with Rubin's rules used to pool estimates (11). Continuous variables were standardized to mean zero and unit variance.

**Figure 1:**
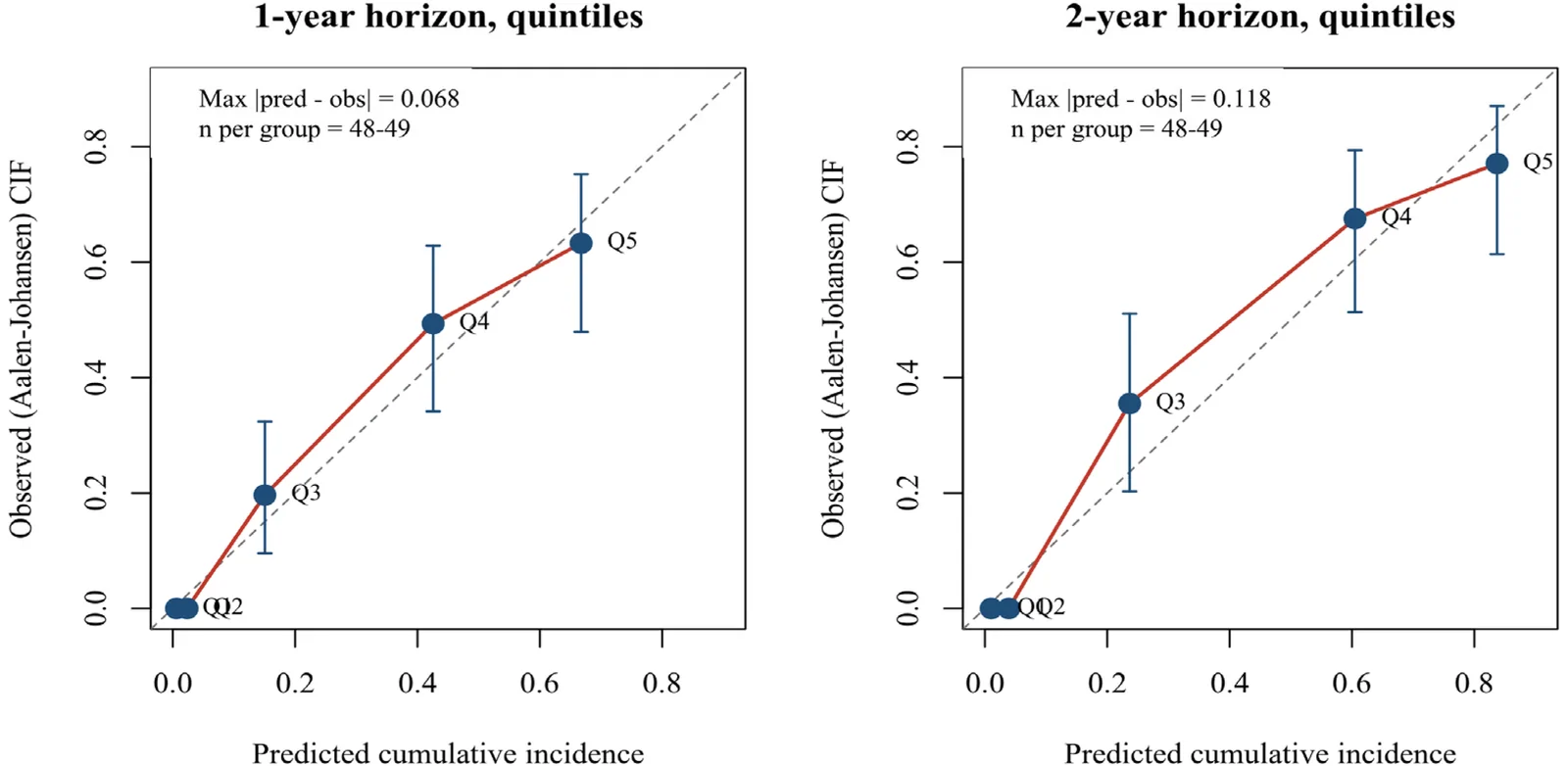
Calibration of predicted vs. observed cumulative incidence of re-failure at 1 and 2 years, by quintile of predicted risk. Points show the mean predicted cumulative incidence and the Aalen–Johansen observed cumulative incidence within each quintile; error bars are 95% confidence intervals for the observed values; the dashed line is the line of perfect calibration.

Discrimination was quantified by the C-index with optimism correction based on 500 bootstrap resamples (12). Calibration was assessed by stratifying patients into quintiles of predicted 1-year cumulative incidence and comparing the mean predicted cumulative incidence with the Aalen–Johansen estimate of the observed cumulative incidence within each group, at 1 and 2 years (Supplementary Table S9 and Figure 1). Decile-based results are provided as a sensitivity analysis in Supplementary Figure S4, Table S10. Firth penalized likelihood was used where rare outcome combinations produced quasi-complete separation.

Prespecified sensitivity analyses included: cause-specific Cox regression; complete-case analysis excluding patients with missing CAC; lesion length dichotomized at the conventional >2 cm threshold; log-transformed CAC as a continuous variable; binary CAC (>0 vs. 0); and exclusion of events within 90 days of PTA. All analyses were performed in R version 4.2.2 using the survival, cmprsk, rms, and mice packages. Two-sided *P* < 0.05 was considered statistically significant.

## Results

### Baseline characteristics

The final cohort comprised 243 patients. During a median follow-up of 18.4 months (IQR, 9.2–31.6), 103 patients (42.4%) experienced re-failure, 24 (9.9%) experienced competing events, and 9 (3.7%) were lost to follow-up. The remaining 107 patients (44.0%) were censored alive with functional fistula.

Table 1 summarizes baseline characteristics by outcome status. Patients who experienced re-failure had markedly longer lesions (median 4.60 cm vs. 0.65 cm) and a substantially higher prevalence of composite stenosis (44.7% vs. 7.1%). Standardized mean differences (SMD) confirmed pronounced imbalance for lesion length (SMD = 2.03) and composite location (SMD = 0.86), with moderate imbalance for anastomotic (SMD = 0.47) and venous outflow (SMD = 0.32) locations. In striking contrast, systemic markers were well balanced: age (SMD = 0.10), diabetes (SMD = 0.11), and CAC score (SMD = 0.14) all fell below the conventional 0.15 threshold for meaningful imbalance.

**Table 1:** Baseline characteristics of the study cohort by outcome status. Values are mean (SD), median (IQR), or n (%). SMD, standardized mean difference; CAC, coronary artery calcium.

Characteristic	Overall	Primary event	No primary event	Missing, *n*	SMD
Age, years	65.15 (11.86)	65.83 (12.42)	64.65 (11.45)	0	0.1
Diabetes	123 (50.6)	49 (47.6)	74 (52.9)	0	0.11
Lesion length, cm	1.75 (0.52–4.37)	4.60 (2.87–6.42)	0.65 (0.26–1.46)	0	2.03
Location: Anastomotic/peri-anastomotic	92 (37.9)	29 (28.2)	63 (45.0)	0	0.86
Location: Arterial inflow	29 (11.9)	9 (8.7)	20 (14.3)	0	0.36
Location: Venous outflow	67 (27.6)	20 (19.4)	47 (33.6)	0	0.17
Location: Composite	55 (22.6)	45 (43.7)	10 (7.1)	0	0.32
CAC score	39.59 (0.00–454.16)	77.72 (3.28–555.92)	17.64 (0.00–434.70)	8	0.14

**Table 2:** Fine-Gray subdistribution hazard model for re-failure after percutaneous transluminal angioplasty. SHR, subdistribution hazard ratio; CI, confidence interval; RCS, restricted cubic spline; CAC, coronary artery calcium.

Variable	SHR (95% CI)	*P* value
Lesion length (restricted cubic spline)		
Lesion length, spline term 1	8.85 (5.98–13.10)	<0.001
Lesion length, spline term 2	0.02 (0.01–0.05)	<0.001
Overall association (2 df)	—	<0.001
Test for non-linearity (1 df)	—	<0.001
Lesion location (ref. anastomotic/peri-anastomotic)		
Arterial inflow	1.18 (0.57–2.41)	0.659
Venous outflow	1.24 (0.70–2.19)	0.460
Composite	1.83 (1.13–2.97)	0.014
Systemic covariates		
Age, per 1-SD increase	0.99 (0.73–1.36)	0.958
Diabetes mellitus	0.97 (0.62–1.52)	0.885
CAC 1–100 vs. 0	1.30 (0.71–2.37)	0.397
CAC >100 vs. 0	1.05 (0.51–2.17)	0.898

### Main Fine-Gray model

**Figure 2:**
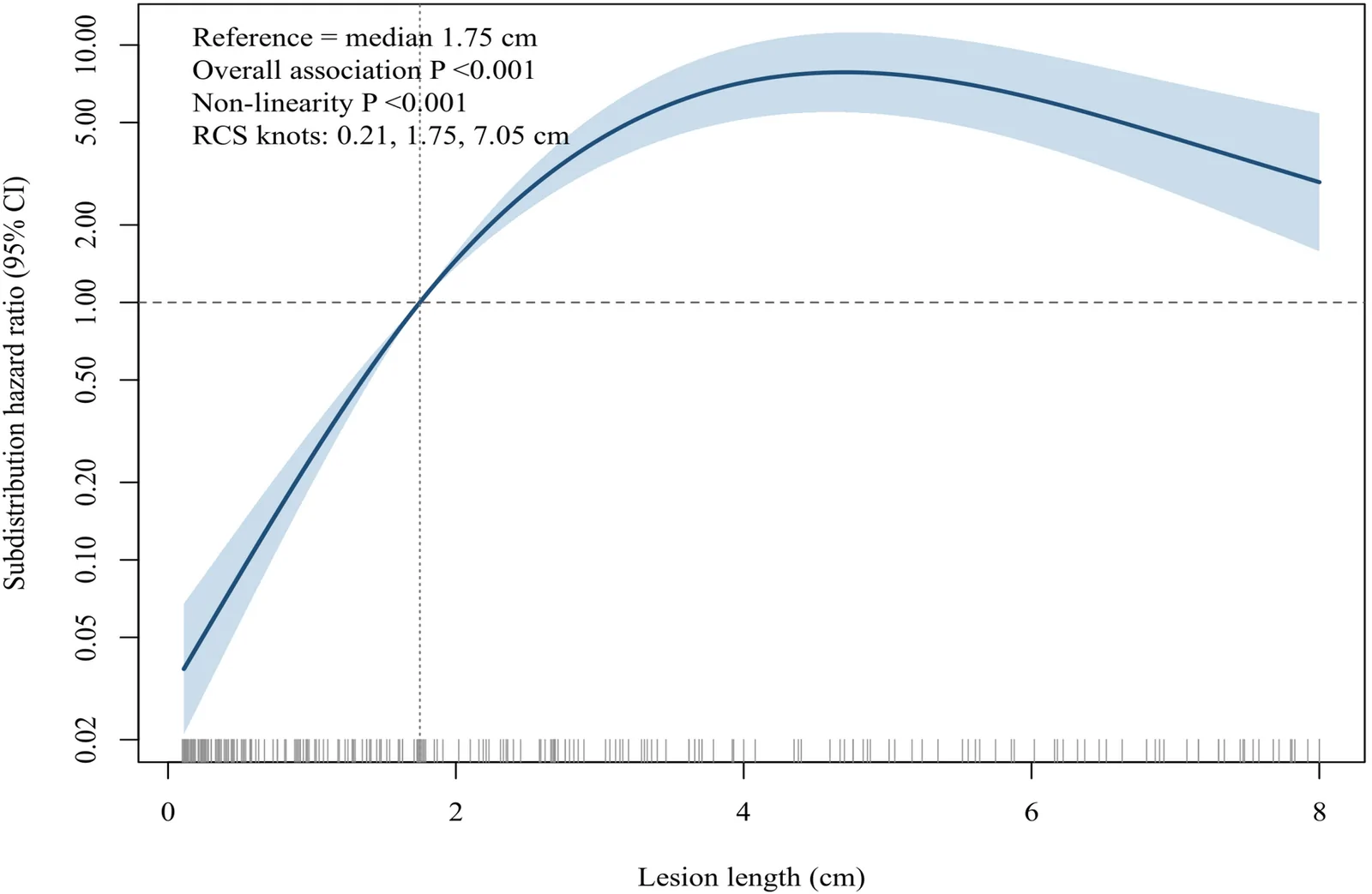
Association between lesion length and re-failure after PTA, modelled with a restricted cubic spline with three knots (0.21, 1.75, 7.05 cm) and referenced at the median length of 1.75 cm. The solid line is the subdistribution hazard ratio and the shaded area its 95% confidence interval; the rug at the bottom shows the observed distribution of lesion length.

In the multivariable Fine-Gray model adjusting for age, diabetes and CAC category, lesion length was strongly associated with re-failure and the association was markedly non-linear (Wald test for non-linearity, chi-square = 91.2, df = 1, *P* < 0.001; overall association *P* < 0.001) (Table 2). Relative to the median length of 1.75 cm, the subdistribution hazard ratio was 0.25 (95% CI, 0.20–0.32) at 1 cm, 1.46 (95% CI, 1.37–1.56) at 2 cm, 4.31 (95% CI, 3.37–5.51) at 3 cm and 7.73 (95% CI, 5.36–11.14) at 5 cm, with the curve flattening beyond approximately 5 cm (Figure 2, Supplementary Table S12). With lesion length modelled in this way, composite morphology remained associated with re-failure but was considerably weaker than under a linear specification: SHR 1.83, 95% CI, 1.13–2.97, *P* = 0.014, compared with 2.81 (95% CI, 1.70–4.66) in the linear model. Neither venous outflow (SHR, 1.24, 95% CI, 0.70–2.19, *P* = 0.460) nor arterial inflow stenosis (SHR, 1.18, 95% CI, 0.57–2.41, *P* = 0.659) was significantly associated with re-failure.

**Figure 3:**
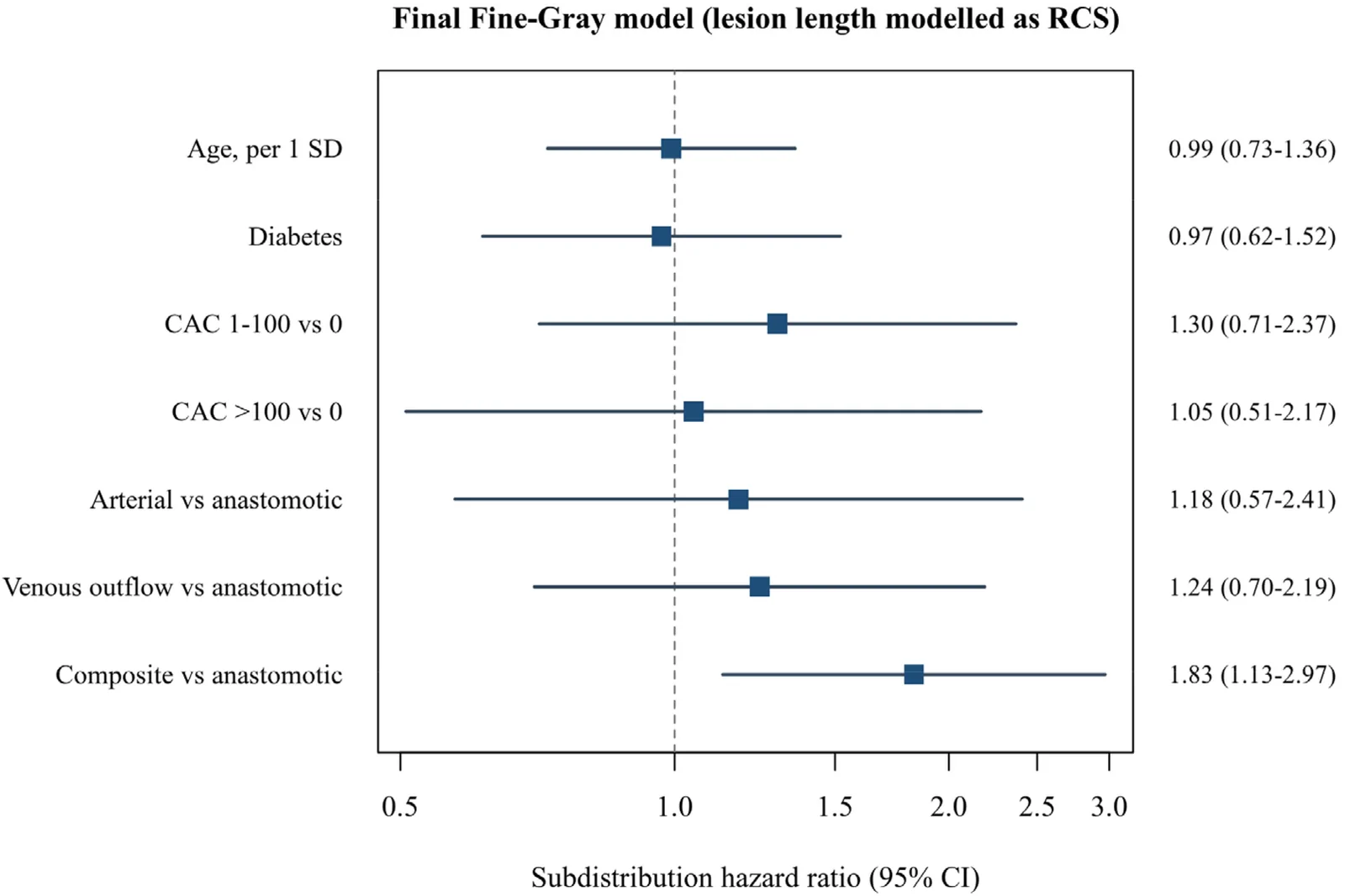
Forest plot of the final Fine-Gray sub distribution hazard model. Lesion length was entered as a restricted cubic spline and is therefore not represented by a single hazard ratio; its association with re-failure is shown in Figure 2.

Conversely, none of the three systemic markers was significantly associated with re-failure: age (SHR, 0.99, 95% CI, 0.73–1.36, *P* = 0.958), diabetes (SHR, 0.97, 95% CI, 0.62–1.52, *P* = 0.885), and CAC category (1–100 vs. 0: SHR, 1.30, 95% CI, 0.71–2.37, *P* = 0.397; >100 vs. 0: SHR, 1.05, 95% CI, 0.51–2.17, *P* = 0.898). Figure 3 displays the forest plot of the final model; because lesion length was entered as a spline, it is not represented by a single hazard ratio and is instead shown in Figure 2.

Collinearity between lesion length and composite stenosis was negligible (VIF <2.0 for both). The interaction term was non-significant (*P* = 0.18), supporting additive rather than redundant prognostic information.

### Model diagnostics and functional form

Assessment of functional form revealed strong evidence of non-linearity for lesion length (chi-square = 91.2, df = 1, *P* < 0.001), whereas age (*P* = 0.222) and CAC score (*P* = 0.150) showed no significant departure from linearity; lesion length was therefore modelled with a restricted cubic spline in all primary analyses. Modelling lesion length as a spline rather than a linear term increased the apparent C-index from 0.783 to 0.807 at 1 year. Sensitivity to the number of knots is shown in Supplementary Figure S6; a 3-knot specification was retained because the 4-knot model was unstable at the lower end of the length distribution and the 5-knot model was not estimable. Graphical proportional-hazards diagnostics are shown in Supplementary Figure S7. Proportional hazards diagnostics suggested potential violations for lesion length (covariate-by-log-time slope = 0.166, *P* = 0.036) and the CAC 1–100 vs. 0 contrast (slope = 0.086, *P* = 0.060). Detailed diagnostics are provided in Supplementary Table S1–S3.

### Time-varying coefficient analysis

In the time-varying coefficient model, the baseline coefficient for lesion length yielded an SHR of 0.98 (95% CI, 0.47–2.05, *P* = 0.964), while the interaction with log-time produced an SHR of 1.13 (95% CI, 0.99–1.29, *P* = 0.070). Because this interaction did not reach statistical significance, we conclude there is insufficient evidence to establish a time-varying effect. The point estimate hints at a numerically stronger effect during the first six months, but this pattern should be regarded as hypothesis-generating. The full follow-up average effect (SHR 1.95) remains the more robust and clinically interpretable estimate. Composite stenosis retained strong significance (SHR, 2.88, 95% CI, 1.77–4.67, *P* < 0.001) with no evidence of time-varying behavior (interaction *P* = 0.477).

### Cumulative incidence, calibration, and discrimination

**Figure 4:**
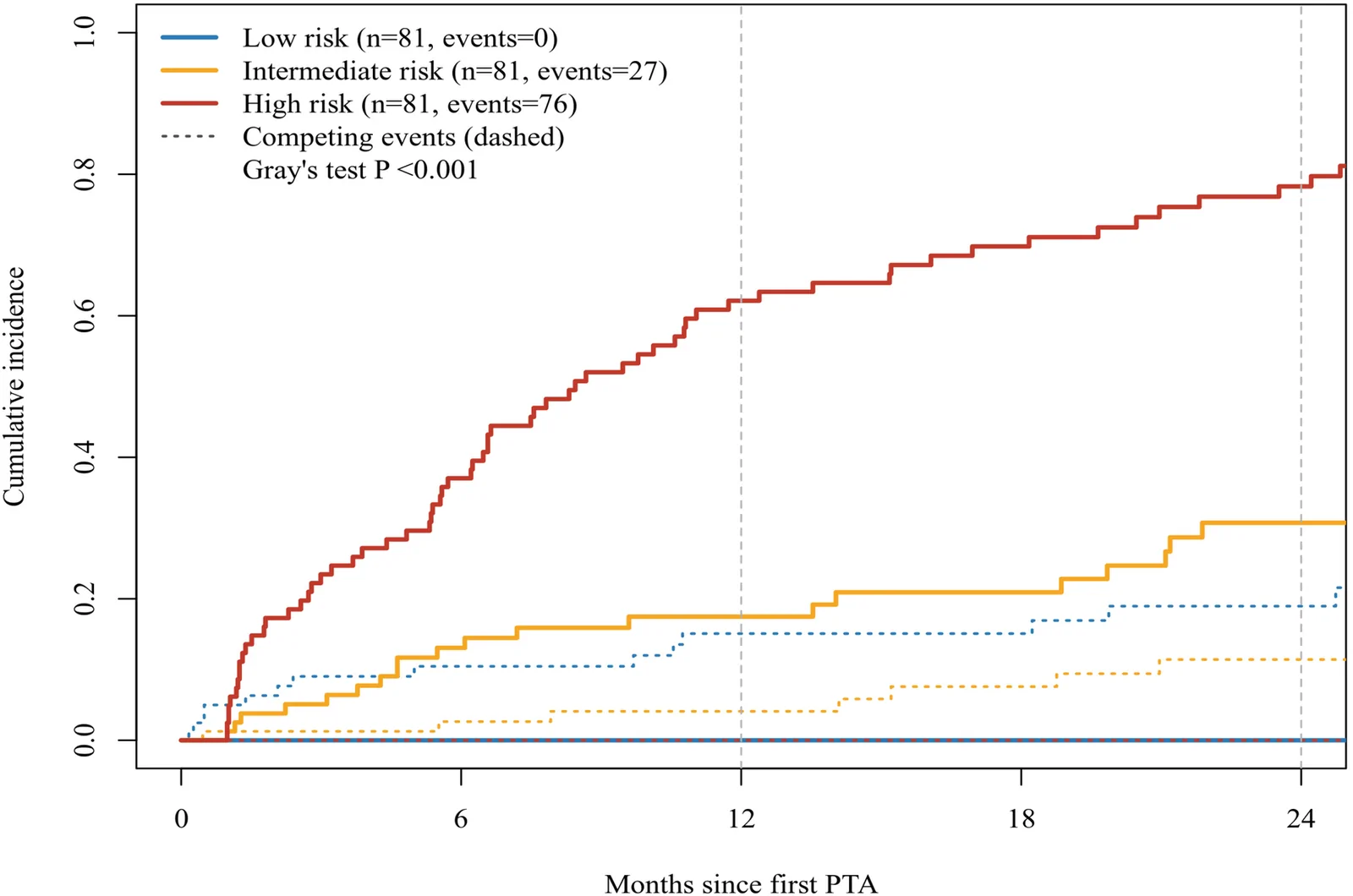
Cumulative incidence of re-failure and of competing events by model-derived risk group. Patients were divided into tertiles of predicted 1-year cumulative incidence (cut-points 0.027, 0.390). Solid lines, re-failure; dashed lines, competing events (death and kidney transplantation); estimates are Aalen–Johansen. Gray's test *P* < 0.001.

Patients were divided into tertiles of model-predicted 1-year cumulative incidence (cut-points 0.027, 0.390), yielding 81 patients per risk group. Cumulative incidence curves separated clearly across these model-derived risk groups (Figure 4; Gray's test chi-square = 133.0, df = 2, *P* < 0.001): the observed 1-year cumulative incidence of re-failure was 0.000 in the low-risk group, 0.175 (95% CI, 0.098–0.270) in the intermediate-risk group and 0.621 (95% CI, 0.504–0.718) in the high-risk group, with corresponding 2-year values of 0.000, 0.307 and 0.783 (Supplementary Figure S2, S3, Table S8). The apparent C-index of the final model was 0.807 at 1 year and 0.812 at 2 years; after correction for optimism estimated from 500 bootstrap resamples, the corresponding values were 0.790 and 0.795 (Supplementary Table S4). Calibration in quintiles of predicted risk (Figure 1) showed acceptable agreement at 1 year, with a maximum absolute deviation between predicted and observed cumulative incidence of 0.068; agreement was poorer at 2 years (maximum deviation 0.118) and in the finer decile-based grouping (0.184 at 1 year and 0.315 at 2 years; Supplementary Figure S4, Table S11), with the largest discrepancies concentrated in the highest-risk groups (Supplementary Figure S5).

### Sensitivity analyses

All prespecified sensitivity analyses supported the robustness of the core findings. Cause-specific Cox models yielded directionally consistent estimates: lesion length HR, 1.85 (95% CI, 1.53–2.25, *P* < 0.001) and composite stenosis HR, 2.75 (95% CI, 1.73–4.38, *P* < 0.001) (Supplementary Table S5). Complete-case analysis produced nearly identical estimates (Supplementary Table S6). When lesion length was dichotomized at >2 cm, quasi-complete separation occurred, yielding an implausibly large SHR, a statistical artifact reinforcing the decision to model lesion length as continuous. Additional analyses using log-transformed CAC, binary CAC, and exclusion of events within 90 days of PTA left the significance and magnitude of anatomical predictors essentially unchanged (Supplementary Table S7). Finally, the estimated effect of composite morphology depended strongly on how lesion length was modelled: the subdistribution hazard ratio for composite lesions was 3.80 (95% CI, 2.33–6.20) when lesion length was omitted from the model, 2.81 (95% CI, 1.70–4.66) with length entered linearly, 2.48 (95% CI, 1.50–4.11) with log-transformed length and 1.83 (95% CI, 1.13–2.97) with the 3-knot spline used in the primary analysis (Supplementary Table S14). This monotone attenuation indicates that a substantial part of the apparent effect of composite morphology is attributable to the greater length of composite lesions.

## Discussion

### Main findings

Our study demonstrates that in grassroots dialysis patients uniformly treated with plain-balloon PTA, lesion length measured on the routine intraoperative angiogram is strongly associated with subsequent re-failure, and that this association is markedly non-linear: risk rises steeply between approximately 1 and 4 cm and changes little thereafter. Composite morphology, which appeared to triple the subdistribution hazard when length was modelled linearly, was attenuated by 35% once length was modelled flexibly, indicating that the two variables are not fully independent and that morphology partly encodes lesion extent. Critically, these predictors require no additional testing beyond the DSA already performed during the index procedure. Only standardized, quantitative reading of existing images is needed, an immediate resource advantage where advanced biomarker platforms or serial imaging are unavailable (8).

### Overlap between lesion length and composite morphology

Because composite stenosis frequently spans multiple segments, we explicitly examined whether the two variables carry overlapping information. Although collinearity diagnostics were unremarkable (VIF <2.0) and the interaction term was non-significant (*P* = 0.18), these tests address linear dependence rather than shared prognostic content. Composite lesions were substantially longer than lesions at other sites (median 4.00 cm vs. 1.63 cm for anastomotic, 0.96 cm for arterial inflow and 1.23 cm for venous outflow lesions; Kruskal–Wallis *P* < 0.001; Supplementary Figure S8), and 83.6% of composite lesions exceeded 2 cm. Consistent with this overlap, the effect attributed to composite morphology fell by 35% once length was modelled as a spline (Supplementary Figure S9), indicating that the two variables should be interpreted as partially redundant rather than additive. Pathophysiologically, this distinction may reflect different disease mechanisms. Long, continuous lesions likely represent diffuse intimal hyperplasia driven by sustained hemodynamic stress and uremia-mediated vascular remodeling (13). Composite skip lesions, where discrete stenotic segments are separated by intervening normal vessel, may signify multifocal hemodynamic disturbance or geographically heterogeneous biological responses to the same systemic milieu; however, this remains a hypothesis requiring dedicated investigation. Their statistical independence justifies simultaneous inclusion in a parsimonious prediction rule.

### Interpretation of negative systemic markers

We caution against interpreting the null results for age, diabetes, and CAC as proof that systemic factors are irrelevant. Given the sample size of 243 patients, these null results may partly reflect Type II error, i.e., insufficient statistical power to detect modest hazard ratios, rather than definitive evidence that systemic factors are unimportant compared with local anatomy. A more plausible explanation is that the anatomical signal was sufficiently strong to dominate weaker systemic effects, and our sample size lacked power to detect modest hazard ratios. Indeed, the literature on diabetes and AVF patency is notably inconsistent: some single-center studies report higher failure rates among diabetic patients (14), whereas larger registries and meta-analyses have repeatedly failed to confirm independent predictive value after adjusting for anatomical and procedural factors (15, 16). Systematic reviews consistently demonstrate that anatomical factors outweigh patient-level comorbidities in predicting short-term patency after intervention (17, 18).

From a pragmatic standpoint, these findings carry an operational message: if systemic markers add little discriminative value beyond what is visible on the intraoperative angiogram, initial post-PTA risk stratification can reasonably focus on local anatomy without extensive laboratory or imaging workup. This parsimony aligns with grassroots practice, where each additional test imposes financial and logistical burden.

### Time-varying effects: exploratory, not confirmatory

The time-varying coefficient analysis produced a borderline interaction term (*P* = 0.070) suggesting that lesion length's effect might strengthen during the first six months. We explicitly caution against overinterpreting this pattern. The interaction did not cross the conventional threshold for significance, and the confidence interval included unity. While the point estimate is compatible with a biologically plausible early high-risk window, during which aggressive neointimal hyperplasia is most active, the current data are insufficient to establish a definitive temporal dynamic. The full follow-up average effect (SHR 1.95) remains the most robust estimate. Future studies with larger samples and more frequent early follow-up may better test this hypothesis.

### Competing event definitions

We treated death and transplantation as competing events because they preclude observation of re-failure. We did not include permanent fistula abandonment as a competing event because, in routine practice, abandonment is almost always a consequence of confirmed or imminent re-failure. Treating abandonment as a competing event would create circular logic: the decision to abandon access is triggered by the very outcome we aim to predict. Our approach prioritizes etiological clarity, consistent with prior vascular access outcomes research.

### Generalizability and device context

The uniform use of Mustang™ plain balloons eliminates device-type confounding but severely limits direct extrapolation to centers routinely using drug-coated balloons (DCBs), covered stents, or cutting balloons, or scoring balloons. In contemporary endovascular practice, these advanced devices are increasingly standard; therefore, the generalizability of our findings to modern treatment strategies is limited. Randomized evidence from meta-analyses demonstrates that DCB angioplasty improves target-lesion patency compared with plain balloon angioplasty (19, 20). Nevertheless, anatomical severity, including lesion length and tandem stenosis, remains prognostically relevant even in advanced-device environments, and the persistently high recurrence burden after PTA underscores the clinical need for better risk stratification regardless of device type (19–21).

### Limitations and future directions

Several limitations merit acknowledgment. First, the single-center retrospective design is susceptible to selection bias, ascertainment bias, and unmeasured confounding. Second, the sample size of 243 provides limited power to detect modest systemic marker effects; null results may partly reflect Type II error. Third, quasi-complete separation with dichotomized lesion length underscores the importance of continuous modeling. Fourth, DSA measurements were performed by the operating surgeon without independent blinded second-reader validation; interobserver measurement error cannot be quantified. Fifth, perioperative antiplatelet and anticoagulant protocols were at physician discretion, introducing potential unadjusted confounding. In the revised analysis lesion length was modelled with a restricted cubic spline rather than a linear term, which addresses the distortion at the extremes of the length distribution but does not resolve a more fundamental issue: discrimination was carried almost entirely by lesion length. The apparent 1-year C-index was 0.700 for lesion location alone, 0.792 for lesion length alone, 0.810 for the two combined and 0.807 for the full model including age, diabetes and CAC (Supplementary Figure S10, S11, Table S13); neither morphology nor the systemic markers added meaningfully once length was known. Sixth, the uniform use of plain non-coated balloons (Mustang™) ensures procedural homogeneity but limits the direct generalizability of this two-variable model. In contemporary endovascular practice, drug-coated balloons (DCBs) have become increasingly standard for maintaining target-lesion patency, and cutting or scoring balloons are frequently employed for resistant lesions. The predictive value of lesion length and composite morphology in the context of these modern devices remains uncertain. Clinicians in centers routinely utilizing advanced endovascular technologies should interpret our findings with caution and consider this model as a reference framework rather than a directly applicable prediction tool until validated in DCB-treated cohorts. Seventh, we defined PTA technical success as residual stenosis <30%, an accepted anatomical threshold. However, this binary categorization inherently groups patients with 0% residual stenosis and those with 29% residual stenosis into the same baseline risk category. From a physiological standpoint, the difference in shear stress and the subsequent trigger for neointimal hyperplasia between a perfectly dilated vessel and one with mild-to-moderate residual stenosis is likely significant. While pre- and post-PTA flow volume data were not systematically available in this retrospective cohort, we acknowledge that this hemodynamic oversimplification represents a limitation. The variance within the “<30% residual stenosis” group could inherently affect re-failure outcomes, and future studies incorporating hemodynamic assessment may refine risk stratification beyond purely anatomical thresholds. Eighth, the proposed model relies on lesion length and composite morphology, but other lesion characteristics that may influence restenosis, including vessel diameter, stenosis severity (percent diameter reduction), local calcification at the target lesion, and thrombotic presentation, were not evaluated. Consequently, lesion length may partly serve as a surrogate marker for overall lesion complexity rather than a purely independent predictor. Future studies incorporating comprehensive lesion characterization are needed to disentangle the independent contribution of each anatomical feature. Ninth, while we evaluated coronary artery calcium (CAC) score as a systemic marker, we did not assess calcification of the target AVF lesion itself. We further verified that the coronary artery calcium score cannot serve as a proxy for local lesion calcification in this cohort: CAC score was not correlated with lesion length (Spearman rho = 0.105, *P* = 0.109, *n* = 235) and did not differ across lesion locations (Kruskal–Wallis *P* = 0.510; Supplementary Figure S12). In dialysis populations, local vascular calcification is an important determinant of balloon expansion adequacy and recurrent stenosis. The absence of local calcification assessment represents an important unmeasured confounder that may influence both procedural success and long-term patency. Tenth, due to the retrospective design and the absence of a standardized procedural data collection form, important procedural variables, including balloon size, inflation pressure, inflation duration, quantitative residual stenosis, and adjunctive thrombectomy, were not systematically recorded. These factors could substantially influence long-term patency and represent an unadjusted confounding source. Prospective studies with standardized procedural documentation are needed to address this limitation.

Arterial calcification is common in end-stage renal disease, yet its correlation with local neointimal hyperplasia in AVF remains uncertain; vascular calcification in dialysis patients is driven by smooth muscle cell apoptosis and osteogenic transdifferentiation, processes that may not directly parallel the intimal hyperplasia dominant in AVF stenosis (21). Current guidelines emphasize integrating local anatomical assessment with systemic risk evaluation (22).

Future research should prospectively validate this two-variable model in multicenter cohorts with heterogeneous device environments. Dual-reader DSA assessment with formal interobserver reliability testing would strengthen measurement validity. Integration of additional anatomical variables, minimum luminal diameter, post-PTA residual stenosis, and vessel curvature, may incrementally improve discrimination. Comparative effectiveness studies evaluating surveillance strategies stratified by DSA-based risk categories against standard monitoring would inform evidence-based guidelines. Finally, cost-effectiveness analyses are needed to quantify the economic impact of adopting zero-cost anatomical risk stratification in resource-constrained dialysis programs.

## Conclusion

A prognostic model based on intraoperative DSA anatomy achieved adequate discrimination (optimism-corrected C-index 0.79 at 1 year and 0.80 at 2 years) in this cohort of grassroots dialysis patients treated uniformly with plain-balloon PTA. Lesion length, modelled flexibly, accounted for nearly all of this discrimination, whereas the apparent contribution of composite morphology was substantially attenuated once length was correctly specified. The model requires no testing beyond the index angiogram and may offer a practical, zero-cost reference for post-PTA monitoring in resource-constrained programmes. However, as a single-centre retrospective derivation study without vessel diameter, stenosis severity, local calcification or procedural data, and without external validation, these findings should be regarded as hypothesis-generating.

## Data Availability

The raw data supporting the conclusions of this article will be made available by the authors, without undue reservation.
