# Supplementary material for: Anatomical lesion extent and recurrent failure after balloon angioplasty in arteriovenous fistulas: a competing-risk analysis
Source: Front Cardiovasc Med. 2026 Aug 12;13:1906106. doi: 10.3389/fcvm.2026.1906106 (PMC13507678; doi:10.3389/fcvm.2026.1906106)
Supplement: Supplementary file 1 [file Datasheet1.docx]

Table S1

| Predictor / test | n | P value | Interpretation |
| --- | --- | --- | --- |
| Panel A. Likelihood-ratio screening for non-linearity (linear vs spline term) |  |  |  |
| Age | 243 | 0.222 | approximately linear |
| CAC score | 243 | 0.150 | approximately linear |

Table S2

| term | slope | p | diagnostic |
| --- | --- | --- | --- |
| CAC_1_100 | 0.0860394969545534 | 0.060378063756294 | crr residual trend vs log(time), first imputed dataset |
| CAC_gt_100 | 0.0072537907433801 | 0.877290370339849 | crr residual trend vs log(time), first imputed dataset |
| age_z | -0.008131512673331 | 0.934052522640078 | crr residual trend vs log(time), first imputed dataset |
| diabetes | 0.0630615439923222 | 0.195784878412672 | crr residual trend vs log(time), first imputed dataset |
| lesion_length_z | 0.166195144927618 | 0.0361981421703241 | crr residual trend vs log(time), first imputed dataset |
| loc_composite | -0.039033283266407 | 0.43615588051258 | crr residual trend vs log(time), first imputed dataset |
| loc_vein_outflow | -0.0219565710806857 | 0.566244687047237 | crr residual trend vs log(time), first imputed dataset |

Table S3

| variable | SHR (95% CI) | P-value |
| --- | --- | --- |
| CAC 1–100 vs 0 | 1.37 (0.74-2.54) | 0.320 |
| CAC >100 vs 0 | 1.32 (0.63-2.74) | 0.460 |
| Age, per 1-SD increase | 0.96 (0.70-1.33) | 0.805 |
| Diabetes | 0.84 (0.54-1.32) | 0.451 |
| Lesion length, per 1-SD increase | 0.98 (0.47-2.05) | 0.964 |
| Composite vs anastomotic/peri-anastomotic | 2.88 (1.77-4.67) | <0.001 |
| Venousoutflow vs anastomotic/peri-anastomotic | 1.43 (0.87-2.36) | 0.158 |
| lesion_length_z_x_logtime*tf1 | 1.13 (0.99-1.29) | 0.070 |

Table S4

| Horizon | Apparent C-index | Optimism | Optimism-corrected C-index | 2.5th–97.5th percentile |
| --- | --- | --- | --- | --- |
| 1 year | 0.807 | 0.017 | 0.790 | 0.784–0.822 |
| 2 year | 0.812 | 0.017 | 0.795 | 0.792–0.825 |

Table S5

| variable | HR (95% CI) | P-value |
| --- | --- | --- |
| CAC 1–100 vs 0 | 1.40 (0.74-2.65) | 0.299 |
| CAC >100 vs 0 | 1.25 (0.59-2.65) | 0.558 |
| Age, per 1-SD increase | 1.00 (0.74-1.34) | 0.988 |
| Diabetes | 0.86 (0.57-1.30) | 0.473 |
| Lesion length, per 1-SD increase | 1.85 (1.53-2.25) | <0.001 |
| Composite vs anastomotic/peri-anastomotic | 2.75 (1.73-4.38) | <0.001 |
| Venousoutflow vs anastomotic/peri-anastomotic | 1.47 (0.84-2.58) | 0.179 |

Table S6

| Model | variable | SHR (95% CI) | P-value |
| --- | --- | --- | --- |
| complete_case | CAC 1–100 vs 0 | 1.44 (0.77-2.69) | 0.253 |
| complete_case | CAC >100 vs 0 | 1.38 (0.67-2.85) | 0.379 |
| complete_case | Age, per 1-SD increase | 0.99 (0.71-1.37) | 0.955 |
| complete_case | Diabetes | 0.89 (0.57-1.39) | 0.618 |
| complete_case | Lesion length, per 1-SD increase | 1.94 (1.60-2.35) | <0.001 |
| complete_case | Composite vs anastomotic/peri-anastomotic | 2.71 (1.69-4.34) | <0.001 |
| complete_case | Venousoutflow vs anastomotic/peri-anastomotic | 1.32 (0.81-2.17) | 0.265 |

Table S7

| Model | variable | SHR (95% CI) | P-value |
| --- | --- | --- | --- |
| abandoned_as_censor | CAC 1-100 vs 0 | 1.39 (0.75-2.58) | 0.299 |
| abandoned_as_censor | CAC >100 vs 0 | 1.34 (0.65-2.75) | 0.431 |
| abandoned_as_censor | Age, per 1-SD increase | 0.98 (0.71-1.35) | 0.880 |
| abandoned_as_censor | Diabetes | 0.85 (0.55-1.32) | 0.471 |
| abandoned_as_censor | Lesion length, per 1-SD increase | 1.95 (1.62-2.35) | <0.001 |
| abandoned_as_censor | Composite vs anastomotic/peri-anastomotic | 2.78 (1.74-4.42) | <0.001 |
| abandoned_as_censor | Venousoutflow vs anastomotic/peri-anastomotic | 1.41 (0.86-2.30) | 0.171 |
| length_gt2 | CAC 1-100 vs 0 | 1.04 (0.56-1.94) | 0.901 |
| length_gt2 | CAC >100 vs 0 | 0.82 (0.41-1.65) | 0.580 |
| length_gt2 | Age, per 1-SD increase | 1.04 (0.78-1.39) | 0.770 |
| length_gt2 | Diabetes | 0.92 (0.60-1.42) | 0.705 |
| length_gt2 | Lesion length >2 cm | 7811654.57 (5551945.09-10991093.41) | <0.001 |
| length_gt2 | Composite vs anastomotic/peri-anastomotic | 2.10 (1.36-3.26) | <0.001 |
| length_gt2 | Venousoutflow vs anastomotic/peri-anastomotic | 1.67 (0.98-2.83) | 0.058 |
| CAC_log | log(CAC+1), per 1-SD increase | 1.14 (0.80-1.63) | 0.460 |
| CAC_log | Age, per 1-SD increase | 0.90 (0.64-1.25) | 0.528 |
| CAC_log | Diabetes | 0.80 (0.51-1.25) | 0.328 |
| CAC_log | Lesion length, per 1-SD increase | 1.97 (1.64-2.36) | <0.001 |
| CAC_log | Composite vs anastomotic/peri-anastomotic | 2.74 (1.71-4.37) | <0.001 |
| CAC_log | Venousoutflow vs anastomotic/peri-anastomotic | 1.37 (0.84-2.25) | 0.205 |
| CAC_binary | CAC>0 vs 0 | 1.37 (0.77-2.45) | 0.284 |
| CAC_binary | Age, per 1-SD increase | 0.96 (0.78-1.19) | 0.731 |
| CAC_binary | Diabetes | 0.84 (0.56-1.27) | 0.415 |
| CAC_binary | Lesion length, per 1-SD increase | 1.95 (1.62-2.34) | <0.001 |
| CAC_binary | Composite vs anastomotic/peri-anastomotic | 2.77 (1.73-4.41) | <0.001 |
| CAC_binary | Venousoutflow vs anastomotic/peri-anastomotic | 1.40 (0.86-2.29) | 0.176 |
| exclude_early_90d | CAC 1-100 vs 0 | 2.65 (1.22-5.76) | 0.014 |
| exclude_early_90d | CAC >100 vs 0 | 1.69 (0.68-4.22) | 0.263 |
| exclude_early_90d | Age, per 1-SD increase | 1.05 (0.74-1.48) | 0.784 |
| exclude_early_90d | Diabetes | 1.02 (0.63-1.66) | 0.935 |
| exclude_early_90d | Lesion length, per 1-SD increase | 2.02 (1.63-2.49) | <0.001 |
| exclude_early_90d | Composite vs anastomotic/peri-anastomotic | 2.61 (1.54-4.43) | <0.001 |
| exclude_early_90d | Venousoutflow vs anastomotic/peri-anastomotic | 1.54 (0.86-2.74) | 0.146 |

Table S8

| Risk group | n | Predicted 1-y CIF | Re-failure | Competing | Observed 1-y CIF (95% CI) | Observed 2-y CIF (95% CI) |
| --- | --- | --- | --- | --- | --- | --- |
| Low | 81 | 0.004-0.027 | 0 | 16 | 0.000 (0.000–0.000) | 0.000 (0.000–0.000) |
| Intermediate | 81 | 0.027-0.389 | 27 | 8 | 0.175 (0.098–0.270) | 0.307 (0.196–0.426) |
| High | 81 | 0.393-0.847 | 76 | 0 | 0.621 (0.504–0.718) | 0.783 (0.670–0.861) |

Table S9

| Quintile | n | Re-failure, n | Competing, n | Predicted CIF | Observed CIF (95% CI) | Absolute difference |
| --- | --- | --- | --- | --- | --- | --- |
| 1 year horizon |  |  |  |  |  |  |
| Group 1 | 49 | 0 | 11 | 0.006 | 0.000 (0.000–0.000) | 0.006 |
| Group 2 | 48 | 0 | 7 | 0.023 | 0.000 (0.000–0.000) | 0.023 |
| Group 3 | 49 | 16 | 6 | 0.151 | 0.196 (0.096–0.324) | 0.046 |
| Group 4 | 48 | 41 | 0 | 0.426 | 0.493 (0.341–0.628) | 0.068 |
| Group 5 | 49 | 46 | 0 | 0.668 | 0.633 (0.479–0.752) | 0.035 |
| 2 year horizon |  |  |  |  |  |  |
| Group 1 | 49 | 0 | 11 | 0.010 | 0.000 (0.000–0.000) | 0.010 |
| Group 2 | 48 | 0 | 7 | 0.039 | 0.000 (0.000–0.000) | 0.039 |
| Group 3 | 49 | 16 | 6 | 0.237 | 0.355 (0.203–0.511) | 0.118 |
| Group 4 | 48 | 41 | 0 | 0.605 | 0.675 (0.513–0.793) | 0.070 |
| Group 5 | 49 | 46 | 0 | 0.837 | 0.771 (0.614–0.870) | 0.066 |

Table S10

| Decile | n | Re-failure, n | Competing, n | Predicted CIF | Observed CIF (95% CI) | Absolute difference |
| --- | --- | --- | --- | --- | --- | --- |
| 1 year horizon |  |  |  |  |  |  |
| Group 1 | 25 | 0 | 8 | 0.005 | 0.000 (0.000–0.000) | 0.005 |
| Group 2 | 24 | 0 | 3 | 0.007 | 0.000 (0.000–0.000) | 0.007 |
| Group 3 | 24 | 0 | 3 | 0.012 | 0.000 (0.000–0.000) | 0.012 |
| Group 4 | 24 | 0 | 4 | 0.035 | 0.000 (0.000–0.000) | 0.035 |
| Group 5 | 25 | 0 | 5 | 0.084 | 0.000 (0.000–0.000) | 0.084 |
| Group 6 | 24 | 16 | 1 | 0.220 | 0.383 (0.189–0.576) | 0.164 |
| Group 7 | 24 | 17 | 0 | 0.369 | 0.307 (0.133–0.502) | 0.062 |
| Group 8 | 24 | 24 | 0 | 0.482 | 0.667 (0.432–0.822) | 0.184 |
| Group 9 | 24 | 22 | 0 | 0.585 | 0.583 (0.356–0.755) | 0.002 |
| Group 10 | 25 | 24 | 0 | 0.747 | 0.680 (0.450–0.830) | 0.067 |
| 2 year horizon |  |  |  |  |  |  |
| Group 1 | 25 | 0 | 8 | 0.008 | 0.000 (0.000–0.000) | 0.008 |
| Group 2 | 24 | 0 | 3 | 0.012 | 0.000 (0.000–0.000) | 0.012 |
| Group 3 | 24 | 0 | 3 | 0.019 | 0.000 (0.000–0.000) | 0.019 |
| Group 4 | 24 | 0 | 4 | 0.059 | 0.000 (0.000–0.000) | 0.059 |
| Group 5 | 25 | 0 | 5 | 0.138 | 0.000 (0.000–0.000) | 0.138 |
| Group 6 | 24 | 16 | 1 | 0.339 | 0.654 (0.386–0.828) | 0.315 |
| Group 7 | 24 | 17 | 0 | 0.540 | 0.496 (0.270–0.687) | 0.043 |
| Group 8 | 24 | 24 | 0 | 0.670 | 0.833 (0.592–0.939) | 0.163 |
| Group 9 | 24 | 22 | 0 | 0.772 | 0.679 (0.434–0.835) | 0.094 |
| Group 10 | 25 | 24 | 0 | 0.899 | 0.856 (0.600–0.954) | 0.043 |

Table S11

| Horizon | Grouping | Maximum absolute deviation |
| --- | --- | --- |
| 1-year | 5 groups | 0.068 |
| 2-year | 5 groups | 0.118 |
| 1-year | 10 groups | 0.184 |
| 2-year | 10 groups | 0.315 |

Table S12

| Lesion length (cm) | SHR (95% CI) vs 1.75 cm |
| --- | --- |
| 0.5 | 0.09 (0.06–0.14) |
| 1.0 | 0.25 (0.20–0.32) |
| 2.0 | 1.46 (1.37–1.56) |
| 3.0 | 4.31 (3.37–5.51) |
| 4.0 | 7.16 (5.15–9.96) |
| 5.0 | 7.73 (5.36–11.14) |
| 6.0 | 6.23 (4.14–9.38) |
| 8.0 | 2.93 (1.58–5.44) |

Table S13

| Model | No. of covariates | Apparent C-index, 1 y | Apparent C-index, 2 y |
| --- | --- | --- | --- |
| length only | 2 | 0.792 | 0.795 |
| location only | 3 | 0.700 | 0.679 |
| length plus location | 5 | 0.810 | 0.813 |
| full model | 9 | 0.807 | 0.812 |

Table S14

| Lesion length specification | Composite SHR (95% CI) | P |
| --- | --- | --- |
| 长度不入模 | 3.80 (2.33–6.20) | <0.001 |
| 线性 | 2.81 (1.70–4.66) | <0.001 |
| log 变换 | 2.48 (1.50–4.11) | <0.001 |
| RCS 3节点 | 1.83 (1.13–2.97) | 0.014 |

Figure S1

Figure S2

Figure S3

Figure S4

Figure S5

Figure S6

Figure S7

Figure S8

Figure S9

Figure S10

Figure S11

Figure S12
